# Consolidated Bioprocessing: Synthetic Biology Routes to Fuels and Fine Chemicals

**DOI:** 10.3390/microorganisms9051079

**Published:** 2021-05-18

**Authors:** Alec Banner, Helen S. Toogood, Nigel S. Scrutton

**Affiliations:** E PSRC/BBSRC Future Biomanufacturing Research Hub, BBSRC/EPSRC Synthetic Biology Research Centre, SYNBIOCHEM Manchester Institute of Biotechnology, Department of Chemistry, School of Natural Sciences, The University of Manchester, 131 Princess Street, Manchester M1 7DN, UK; alec.banner@postgrad.manchester.ac.uk

**Keywords:** lignocellulose degradation, cellulases, biofoundry, consolidated bioprocessing, synthetic biology

## Abstract

The long road from emerging biotechnologies to commercial “green” biosynthetic routes for chemical production relies in part on efficient microbial use of sustainable and renewable waste biomass feedstocks. One solution is to apply the consolidated bioprocessing approach, whereby microorganisms convert lignocellulose waste into advanced fuels and other chemicals. As lignocellulose is a highly complex network of polymers, enzymatic degradation or “saccharification” requires a range of cellulolytic enzymes acting synergistically to release the abundant sugars contained within. Complications arise from the need for extracellular localisation of cellulolytic enzymes, whether they be free or cell-associated. This review highlights the current progress in the consolidated bioprocessing approach, whereby microbial chassis are engineered to grow on lignocellulose as sole carbon sources whilst generating commercially useful chemicals. Future perspectives in the emerging biofoundry approach with bacterial hosts are discussed, where solutions to existing bottlenecks could potentially be overcome though the application of high throughput and iterative Design-Build-Test-Learn methodologies. These rapid automated pathway building infrastructures could be adapted for addressing the challenges of increasing cellulolytic capabilities of microorganisms to commercially viable levels.

## 1. Introduction

Many of the social and technological advances in the last century, from transportation fuels to materials and pharmaceuticals, have been due to an increase in our understanding and utilisation of organic chemistry [1]. Much of this chemistry relies on the use of fossil carbon as synthons and is therefore inextricably coupled to the petrochemical industries. These reactions often require high temperatures, high pressures and rare metal catalysts [1], thereby generating polluting waste. Recognition of a global environmental crisis is in part driven by our over use and reliance on petroleum-based fuels and chemistries [2]. Alternative “green” synthetic routes have been developed, utilising non-fossil fuel-derived renewable biomass as synthons [3,4,5,6,7,8,9,10,11,12,13]. These emerging biotechnologies rely on the microbial conversion of biological carbon biomass (e.g., sugar cane; biomass waste streams) into advanced synthetic fuels and bio-based chemistries [14]. A report into the development of the bio-economy through to 2030 suggests biotechnological routes have the potential to produce 75% of pharmaceutical or 35% of total chemicals currently made via synthetic chemistry [15].

Traditional genetic engineering routes to biocatalytic processes are increasingly being superseded by synthetic biology technology, which employs a fermentative recombinant microbial approach to fine chemical production [5,16,17,18,19,20]. In this case, individual “parts” of the introduced enzyme pathway(s) (e.g., enzyme homologues, promoters and ribosomal binding sites) are optimised to increase the flow through the pathway [21,22,23]. This process is often assisted by computer-aided-design programs to predict the optimal arrangement and sequence of each component [24]. This revolutionary approach allows for the development of de novo pathways to chemicals not found in nature, and can take advantage of enzyme engineering technologies to generate enzymes that catalyse novel reactions [25]. Examples of (bio)compounds produced by engineered microorganisms using a synthetic biology approach include artemisinic acid [26], β-farnesene [27], linalool [17,28,29], noscapine [30], butanol [31], 6-aminocaproic acid [32] and styrene [33]. The most complex to date was the complete synthesis of noscapine in *Saccharomyces cerevisiae*; an antitumor alkaloid derived naturally from *Papever somniferum* (opium poppy) [30]. In this case, eighteen heterologous enzymes were expressed in *S. cerevisiae*, of which only thirteen sequences were obtained from the native poppy.

While the uptake of bio-based synthetic routes is increasing, significant advances are needed to increase the cost-effectiveness of these processes, to enable them to compete commercially with existing synthetic chemical or native biological routes [34]. As a result, few biosynthetic routes have reached industrial commercialisation, largely due to low product yields and the high cost of feedstocks. The largest scale commercial bioproduct is bioethanol produced from *S. cerevisiae* [35], with 29,000 million gallons generated worldwide in 2019 [36]. Most bioethanol is produced through anaerobic fermentation of glucose derived from either corn or sugarcane [37,38]. However, both crops are in direct competition with land use for food production. In a world where deforestation and famine are major issues, this has led some people to declare these fuels of little benefit compared to traditional fossil fuels [39].

A more environmentally sustainable solution is the utilisation of waste plant biomass or lignocellulose waste. Each year, around 200 billion tonnes of lignocellulosic waste are produced by industries such as farming and agriculture [40], and have limited commercial value. Typically, this waste would either be combusted, composted or used as a bulking agent in animal feed. The utilisation of this waste in synthetic biology applications could add commercial value to the waste and provide a carbon neutral source of fuels and other high value compounds. However, existing commercial microbial fermentations utilising lignocellulose waste as a carbon source rely on the release of the abundant recalcitrant sugars (e.g., glucose) via expensive pre-treatment strategies [41]. 

An alternative approach could be to employ a consolidated bioprocessing (CBP) strategy, whereby biocatalytic enzyme production, lignocellulose degradation (saccharification) and fermentation are accomplished within a single microorganism. This approach would likely reduce feedstock pre-processing requirements (and associated costs), making a more industrially viable and “green” process. To achieve this, either existing commercial strains require engineering to incorporate an extracellular localising cellulolytic system, or secondary product biocatalytic pathways need to be integrated into naturally cellulolytic microorganisms. 

This review discusses current approaches and challenges for the utilisation of waste lignocellulose biomass as a feedstock for building a robust microbial chassis which can produce high value biomaterials, thereby enabling “green” solutions to biochemical production, and be competitive with chemical synthesis. We later propose the future application of tools developed by the rapidly expanding application of biofoundries, which have had a significant impact on the production of biosynthetic pathways, to the challenges of producing novel, cellulolytic biofactories.

## 2. Lignocellulose as a Carbon Source

### 2.1. Lignocellulose: A Heterogeneous Source of Polymeric Sugars

Lignocellulose is potentially an ideal target as a low-cost carbon and energy source for microorganisms as it is the most abundant biologically derived polymer found in nature [14]. It is composed of an intricate species-specific network of cellulose (40–50%), hemicellulose (20–40%) and lignin (20–35%). The hemicellulose interweaves with cellulose polymers, while the lignin content protects the cellulose from degradation [42]. The compact and intertwining nature of the individual polymer types in lignocellulose makes it a multifaceted and challenging task for enzymatic degradation. 

**Figure 1:**
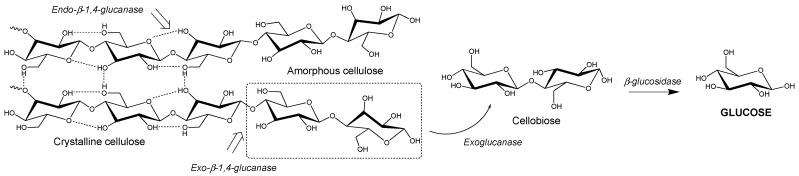
Enzymatic degradation of cellulose to glucose.

The major saccharification target is cellulose, a polysaccharide composed β-1,4 linked D-glucose (Figure 1) [43]. This polysaccharide can pack together using a network of hydrogen bonding (i.e., “crystalline” cellulose) to form tightly packed microfibrils, which are difficult to be degraded by enzymes [43]. This is due to the difficulty of lignocellulose-degrading enzymes to gain access to the majority of the glucose monomers when it is in the crystalline state. Therefore, lignocellulose usually undergoes thermochemical or similar pre-treatment strategies prior to enzymatic saccharification to remove the hemicellulose and lignin, and decrease the crystallinity of the cellulose fibres. As glucose is the most widely accepted carbon source for microorganisms [44], unlocking this recalcitrant cellulose to release the abundant glucose molecules makes lignocellulose a potentially rich feedstock. 

**Figure 2:**
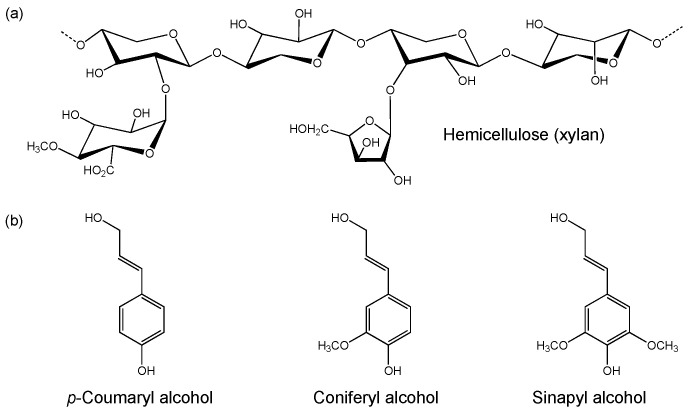
Example structures of (**a**) hemicellulose (xylan) and (**b**) monomers of lignin.

Hemicellulose is a heterogenous polysaccharide that is comprised of a diverse array of C5 and C6 sugar monomers. It generally contains a xylan (major component), galacto(gluco)mannan or xyloglucan backbone [45] with branching side chains (Figure 2a) [46]. Differences in hemicellulose composition are seen between plant species, including the range of sugar and sugar acid classes present and their linkage types. These monomeric units include D-xylose, D-mannose, D-galactose, D-glucose, L-arabinose, 4-O-methyl glucuronic acid, D-galacturonic and D-glucuronic acids [42]. The monomers are linked via β-1,4- and β-1,3-glycosidic bonds. Given the diversity in hemicellulose composition, efficient degradation requires a broad range of hemicellulases compared to cellulose breakdown [47]. Hemicellulose is considered to be of lower value as a carbon source compared to cellulose due to the presence of C5 sugars, which are often not degraded by microorganisms [48].

Lignin is a complex heteropolymer composed of units of phenylpropane derivatives, such as *p*-coumaryl-, coniferyl- and sinapyl alcohols (Figure 2b) [42]. These compounds are linked via C–C and C–O bonds, and form *p*-phenyl- (H type), guaiacyl- (G type) and syringyl (S type) structural monomers. Lignin is covalently linked to both cellulose and hemicellulose, and provides the plant with structural support and impermeability. It also functions as a resistance against microbial attack and oxidative stress. Given these characteristics, lignocellulose requires pre-treatment to remove lignin to release the cellulose prior to enzymatic saccharification. Lignin is generally not considered to be a target carbon source for microorganism cultivation, but instead is a source of valuable phenolic synthons for the production of high-value compounds [49,50]. 

### 2.2. Lignocellulose Pre-Treatments

Currently, most commercial and pilot scale processes utilising lignocellulose as a feedstock require physical and/or chemical pre-treatments to remove hemicellulose and lignin, reduce the crystallinity of the cellulose and minimise the release of hemicellulose-derived inhibitory compounds (e.g., furfural). The resultant amorphous regions of the cellulose then undergo enzymatic hydrolysis by commercial cocktails of cellulolytic enzymes to release glucose for later fermentations [51]. There are four main classes of lignocellulose pre-treatment strategies tested for their effectiveness in releasing amorphous cellulose with minimal inhibitory compounds. The first are purely physical techniques designed to break down the size of cellulose fibres and degrade lignin and hemicellulose. These techniques include size reduction (chipping, grinding and milling), microwave irradiation, ultrasound and high-pressure homogenisation [41]. These energy-intensive processes successfully reduce the crystallinity of the cellulose, but are generally not commercially viable options.

A second group of pre-treatments are physio-chemical processes, such as steam explosion and hot liquid water treatment [41]. Steam explosion treatment is an effective process, leading to the breakage of the fibres, allowing easy access of enzymes to the cellulose for hydrolysis to occur. It also causes delignification and solubilisation of hemicellulose. However, hemicellulose transformation is incomplete, and toxic compounds are released. Chemical treatments with acids [52], alkalis [53], oxidation agents, biological solvents [54] and aqueous–organic solvents have also been devised with mixed success [41]. They often successfully remove lignin with low inhibitor release, but suffer from high reagent costs and the need for corrosion resistance in scaled equipment. The final class of pre-treatments is purely biological, where cellulolytic microorganisms are used to partially decompose the lignocellulose to break up its structure. Typical microorganisms used include brown, white and soft rot fungi, with higher yields of glucose release after later enzymatic treatments due to increased cellulose purity. The disadvantage of biological treatments is the lower reaction rates, with extended residence times needed for efficient breakdown of lignocellulose [41]. 

Overall, there have been extensive studies on determining the most efficient and cost-effective method for lignocellulose pre-treatment to maximise glucose output for later fermentation [41,55,56,57]. Consideration must be paid to the type of lignocellulose (cellulose vs. hemicellulose content), the potentially high costs involved and the formation of toxic side products which can inhibit subsequent microbial fermentations [58]. The environmental impact must also be considered, such as the high energy usage and harsh chemicals needed in many pre-treatments, which impact on an otherwise “green” process.

### 2.3. Enzymatic Lignocellulose Degradation

More than 160,000 cellulases have been identified [59], which share a general acid/base mechanism of catalysis [60]. These cellulose and hemicellulose degrading enzymes are classified into different families within the CAZy (Carbohydrate-Active Enzymes) database, based on sequence and structural features. Almost all glycosyl hydrolase (GH) cellulolytic enzymes studied to date for commercial utilisation have originated from fungal species, with only a few bacteria examples investigated [61]. The known crystal structures of these enzymes show they typically contain a carbohydrate binding module, which is attached to the catalytic domain via a flexible linker region [42]. In addition, most fungal cellulases have undergone N- and O-glycosylation by post-translational modification. Glycosylation enhances catalytic activity, and increases structural and thermal stability [62]. Bacterial cellulases do not undergo glycosylation, and the functioning of bacterial homologues is less well understood. 

Cellulose is enzymatically degraded to glucose units (C6 sugar) by glycoside hydrolases (cellulases) via the hydrolysis of its β-1,4 glycosidic bonds [60]. The complete degradation of cellulose microfibrils requires the synergistic action of three types of cellulases, namely an endoglucanase, exoglucanase and β-glucosidase (Figure 1) [63]. Endoglucanases randomly cleave β-1,4-glycosidic bonds between glucose monomers within the cellulose chain. They can be either non-processive or processive; the latter allowing several consecutive cleavages on the same polysaccharide chain as the substrate threads through the active site [64]. They are generally most active in the amorphous region of cellulose [65]. Conversely, exoglucanases cleave cellobiose (glucose–glucose unit) from the end of cellulose chains in a processive manner, and are often more active in the crystalline regions of cellulose [66]. Processive exoglucanases are also known as cellobiohydrolases, and are usually the major constituent of natural and commercial cellulase mixtures. Finally, β-glucosidases cleave cellobiose to release two free glucose molecules, which can then be used as a carbon and energy source by microorganisms [65]. Natural cellulolytic microorganisms often contain several different exo- and endo-acting cellulases, to enable them to degrade different forms/faces of cellulose [64].

In addition to classic cellulases, the glycosyl hydrolase family GH61 are known to exhibit “cellulolytic enhancing ability” when combined with common cellulases [67]. For example, TaGH61 from *T. aurantiacus* generates C1 oxidised polysaccharide oligomers from cellulose with a non-reducing end oxidised species. This enzyme enabled an increase in microcrystalline cellulose degradation by other cellulases in the presence of gallic acid. This new class of enzymes are known as copper-dependent lytic polysaccharide monooxygenases (LPMO) [64]. They cleave the glycosidic bond within crystalline regions of the cellulose to produce aldonic acids [64]. Oxidation occurs at the C1 carbon, and possibly also C4 and C6, dependent on the enzyme homologue. This leads to a breaking up of the crystalline regions of cellulose, which greatly enhances the degradation of cellulose by allowing access to traditional cellulases [42].

Due to the complex nature of hemicellulose, sugar release requires the cooperative action of multiple types of enzymes. For xylan degradation, one of the two predominant enzymes required are endo-1,4- β-xylanases, which hydrolyse β-1,4-xylan to yield xylo-oligosaccharides. The second major enzymes are exo-1,4- β-xylosidases, which hydrolyse xylobiose and xylo-oligosaccharides to produce xylose (C5 sugar). Mannan (β-1,4-linked mannose) and glucomannan are major hemicellulose constituents of softwood [42]. Degradation of these C6-sugar polymers requires the action of endo- β-1,4-mannanases, which hydrolyse oligosaccharides with three to four monomers. This is followed by exo- β-1,4-mannosidase, which hydrolyses terminal non-reducing β-mannose residues. For glucomannan cleavage, β-glucosidases cleave the bond between mannose and glucose units in the polymer [42]. 

Additional accessory enzymes are found in natural systems to assist in the efficient hydrolysis of hemicellulose [42]. These enzymes are acetylxylan esterase, feruloyl esterase, *p*-coumaroyl esterase, α-L-arabinofuranosidase, xylan α-1,2-glucuronosidase and α-glucuronidase. However, strategies for the utilisation of lignocellulose as a carbon source usually involve the removal of its hemicellulose content. One would envisage that the inclusion of all eight recombinant hemicellulose-degrading enzymes as well as the three cellulose-degrading enzymes within the target host may not be the most efficient strategy for optimising carbon utilisation.

Commercial cellulase cocktails, produced by companies such as Novozyme, are typically made up of cellulases from *T. reesei* [68], supplemented with additional enzymes [69] such as α-xylosidase [70] or GH5 [71]. The cost of using commercial enzyme cocktails to release free sugars from lignocellulose has been shown to represent up to 48% of the final cost of second-generation bioethanol in some demonstration scale plants [72]. Reducing this cost is therefore essential in the development of future cost-competitive and renewable bio-based processes.

Lignin removal is one of the primary targets of thermochemical lignocellulose pre-processing as it is highly insoluble and can form covalent crosslinks with hemicellulose side chains, conferring additional strength to plant cell walls [73]. The composition of lignin is plant species specific, and is not a readily fermentable carbon source for microorganisms. There are natural enzymes that can degrade lignin, namely laccase, peroxidases, oxidases, aryl-alcohol dehydrogenase, cellobiose dehydrogenase, catechol oxidases and tyrosinases [42]. The exact combination of enzymes and mechanism of degradation varies by microorganism type [74].

### 2.4. Cellulase Localisation

**Figure 3:**
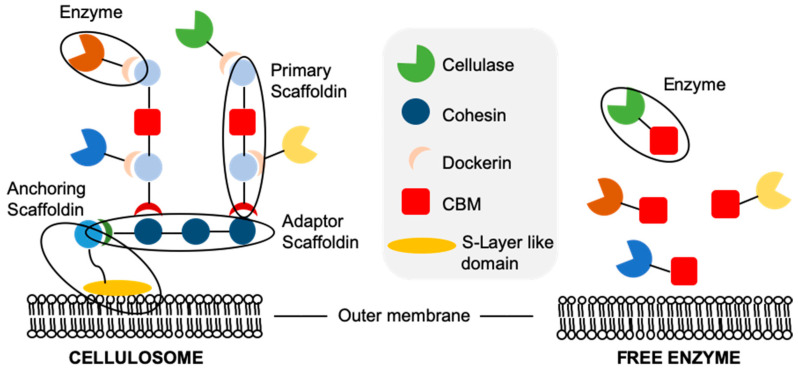
Schematic representation of free cellulases and cellulosomes.

Degradation of the highly insoluble lignocellulose by microorganisms requires that all cellulolytic enzymes must be expressed extracellularly. In naturally cellulolytic microorganisms, the extracellular saccharification machinery exists as either free (secreted) enzymes [75], or associated with the outer membrane in multi-enzyme cellulosomal complexes [76] (Figure 3). The targeting of enzymes into either cellulosomes or as free extracellular enzymes is achieved by the presence of an N-terminal signalling peptide sequence. Aerobic bacteria and fungi tend to secrete multidomain cellulases, such as *Tricoderma reesei* [75]. These enzymes diffuse to and bind lignocellulose, the latter via their carbohydrate binding modules (CBMs). The associated catalytic domain then hydrolyses the substrates, releasing oligosaccharides for later hydrolysis into free sugars [77]. The cellulase CBM domains increase the rate of hydrolysis of lignocellulose by effectively increasing the enzyme concentration around the substrate compared to enzymes containing only a catalytic domain.

Cellulolytic anaerobes, such as *Clostridium thermocellum*, employ cell-associated multi-enzyme cellulosomes composed of all the key hydrolases needed for lignocellulose degradation (Figure 3; [78]). These complexes are formed around proteins called scaffoldins, consisting of multiple cohesin domains and a dockerin domain. The anchoring scaffoldin contains a single, C-terminal, S-layer-like domain which binds peptidoglycans in the microbial cell wall, anchoring the cellulosome to the cell. The hydrolases contain both an active catalytic domain and a second, non-catalytic dockerin domain. These dockerins bind the cohesion domains and effectively target the enzyme within the cellulosome. The CBMs are contained within the primary scaffoldin, and play a role in binding the cell to the cellulosic substrate. 

Variability exists in the exact arrangement between the different protein constituents within cellulosomes; however, the primary roles of the components remain unchanged. Cellulosomal systems generally have a lower lignocellulose hydrolysis rate than free enzyme systems, as they are limited by the upper limit of enzyme surface loading onto the microorganism’s outer membrane [76]. In addition, enzymes displayed on cell surfaces cannot penetrate as deep into lignocellulose as do free enzymes. In spite of this, cellulosomes enable an increase in the localised concentration of free sugars available to the cell [79]. Additionally, cellulases often display synergism with one another, and localisation within a cellulosome may enhance this effect [80]. 

A difficulty encountered with some target bacterial microbial chassis is the poor efficiency of extracellular secretion of recombinant proteins through the outer membrane, whether it be for cell-surface display or as free enzymes. This is especially problematic with Gram-negative bacteria, such as non-pathogenic strains of *E. coli*. These organisms contain an outer membrane lipopolysaccharide bilayer that acts as an effective permeability barrier. Enzymes are secreted via the general secretory (*sec*) or twin-arginine translocation (*tat*) pathways, and typically end up in the periplasmic space separating the two membranes [81]. Extracellular protein secretion is sometimes achieved by inefficient passive transport from the periplasmic space via outer membrane proteins [82]. Whilst secretory pathways are present in Gram-negative bacteria [83], they are often poorly understood and successful extracellular secretion is technically challenging to achieve in many cases. The challenges involved in exporting the required enzymes are one of the biggest challenges faced for the engineering of microorganisms for CBP. A variety of factors can affect the rate of extracellular secretion within *E. coli*. The most frequent problems encountered are incomplete secretion into the periplasmic space, insufficient capacity of the export machinery, and proteolytic degradation of the recombinant proteins [84]. Additional factors influencing secretion efficiency include protein size, leader peptide amino acid composition (sequence) and protein production rates outstripping the maximal secretion rate [84]. 

Gram-positive bacteria, in contrast, can often secrete large amounts of recombinant proteins into the surrounding medium, which makes them attractive microbial chassis for growth on lignocellulose waste. Gram-positive bacteria and fungi have a single cell membrane through which enzymes can be transported via either the *sec* [85] or *tat* pathways [86]. Not all classes of proteins are well secreted, but the efficiency generally outstrips the relatively poor levels seen with Gram-negative bacteria. Efficient secretion can also face bottlenecks of proteolytic cleavage, secretion stress with the associated metabolic burden. Examples of Gram-positive bacteria with proven ability for efficient protein secretion include the genera *Bacillus, Corynebacterium, Streptomyces* and *Lactobacillus*. 

Yeast is a promising microbial host for secondary metabolite production from cells grown on pre-treated lignocellulose. It has the added advantage of containing the cellular machinery required for post-translational glycosylation of enzymes, enabling highly efficient fungal cellulases to be expressed and secreted in an active form. For example, one study described *Saccharomyces cerevisiae* strains which were engineered to secrete both a cellulase and a xylanase for efficient degradation of partly delignified corn stover [87]. The synergistic action of both enzymes increased ethanol titres by up to 3.4-fold compared to wild type *S. cerevisiae*. A second study engineered the *Clostridium thermocellum* scaffoldin gene *CipA* and anchoring protein gene *OlpB* into the industrial yeast *Kluyveromyces marxianus* [88]. This organism expressed a cellulosome containing a mixture of dockerin-fused fungal cellulases, including exoglucanase, β-glucosidase, endoglucanase and accessory cellulase “booster” genes. This enabled growth on phosphoric acid-swollen cellulose, which yielded ethanol titres of 8.61 g/L [88].

## 3. Consolidated Bioprocessing

Consolidated bioprocessing (CBP) is a biomanufacturing approach that combines the saccharification of lignocellulose waste with fermentation to produce the desired compounds within the same microbial chassis [89]. By combining these steps into a single microbial process, there is the potential to reduce the costs associated with the saccharification of pre-treated lignocellulose by eliminating the need to pre-release sugars for fermentation using expensive commercial enzyme cocktails. A successful CBP strategy requires the microorganism to secrete a range of native or recombinant extracellular cellulolytic enzymes in addition to the required pathway enzymes for making the industrially useful secondary product. 

Microbial host selection is critical when designing CBP routes to chemical and advanced synthetic fuel production. Naturally cellulolytic microorganisms are obvious targets, as they contain all the machinery for completely digesting lignocellulose with minimal pre-processing. However, naturally cellulolytic microorganisms may not be the most industrially robust chassis for chemical production, and may require engineering to introduce the pathways to make the desired compound, or improve the natural titres. Alternatively, non-cellulolytic microorganisms which currently produce high yields of the target compounds could be engineered to introduce a secretable cellulolytic system.

### 3.1. Naturally Cellulolytic Microorganisms

Naturally cellulolytic microorganisms are superbly adapted for lignocellulose degradation and subsequent growth compared to de novo engineered bacteria. The major challenge often associated with these organisms is the need to develop rapid and efficient synthetic biology tools to enable the incorporation of pathways necessary to produce high yields of target compounds [89]. This may include non-native pathway incorporation and/or upregulation of cellular precursors and natural (bio)chemical production. 

The main research in this area is looking at improving biofuel titres with the microorganisms *Trichoderma reesei*, *Clostridium cellulolyticum* and *Clostridium thermocellum* grown on lignocellulose. In one study, *T. reesei* CICC 40360 underwent nitrosoguanidine treatment followed by genome shuffling mutagenesis to increase ethanol production. This improved ethanol titres five-fold under aerobic conditions, in addition to enhancing ethanol resistance [90]. The thermophilic anaerobe *C. thermocellum* ATCC 31924 was also investigated for its ethanol production titres when grown on crystalline cellulose. This cellulosome-producing strain under optimised cultivation conditions generated 0.3 g ethanol per gram of cellulose digested, with >95% cellulose conversion [91]. A further 20% increase in ethanol titres was achieved by shifting carbon flux away from lactate production by the inclusion of acetate in the medium [91].

A modified isobutanol pathway was engineered in *C. cellulolyticum* based on the L-valine biosynthetic pathway [92]. This route is based on diverting glucose-derived 2-keto acid intermediates through to isobutanol using recombinant enzymes from *Bacillus subtilis*, *E. coli* and *Lactococcus lactis*. Isobutanol titres of 0.66 g/L were obtained when grown on cellulose, compared to 15–20 g/L from free glucose-based carbon sources [92]. Therefore, increases in the cellulose utilisation rate will likely be needed before this process becomes commercially viable. The thermophilic variant *C. thermocellum* also underwent engineering for isobutanol production [93]. Unfortunately, this strain suffered from enzyme toxicity and other challenges during pathway engineering. Eventually, a stable genomic integrated isobutanol-producing strain was generated, showing isobutanol titres of 5.4 g/L when grown on cellulose at 50 °C (41% of the theoretical yield) [93]. This study highlighted some of the problems encountered when using non-model organisms as microbial chassis with fewer available molecular biology tools. 

### 3.2. Non-Cellulolytic Chemical Producers

The alternative strategy for CBP is to engineer existing microorganisms producing commercially relevant compounds, both native and engineered systems, with a functional extracellular cellulolytic system. This opens up a wider range of possible microbial chassis, and allows us to take advantage of the extensive molecular engineering toolboxes available for model organisms. The incorporation of an efficient cellulolytic system into a new microbial chassis requires additional considerations over biocatalytic pathway engineering, as each enzyme must be either secreted extracellularly or displayed on the outer membrane. 

*Yarrowia lipolytica* is a non-conventional yeast with significant biotechnological potential due to its native ability to produce bio-surfactants, *γ*-decalactone, citric acid, intracellular lipids and lipase [94]. It has undergone multiple engineering studies to increase its hydrolytic secretome to include growth on complex polysaccharides such as starch, cellulose, xylan and inulin. Genome analysis of *Y. lipolytica* revealed the presence of multiple intracellular and extracellular β-glucosidase genes and putative cellobiose transporters, which explained why cellobiose could be assimilated intracellularly, but growth on cellulose was not possible [95]. Growth on pre-treated corn stover was achieved (50%) after engineering in the *T. reesei* cellulase genes *EGII* and *CBHII* [94]. A dormant pathway for xylose utilisation was found in the *Y. lipolytica* genome, but not xylan degradation. Multiple studies engineered xylanase genes into *Y. lipolytica*, including the cell-surface expression of the *XYN* gene from *Thermobacillus xylanilyticus* [96]. Interestingly, the sole expression of XynII from *Trichoderma harzianum* into *Y. lipolytica* enabled growth on birchwood xylan as the sole carbon source [94]. 

The transition from first generation (sugar-starch feedstocks) to second generation (lignocellulose biomass) bioethanol production necessitated the incorporation of secretable saccharolytic machinery into *S. cerevisiae*. In one study, three cellobiohydrolases (*cbh1* from *Aspergillus aculeatus* and *cbh1*/*cbh2* from *Trichoderma reesei*) were integrated into the genome of *S. cerevisiae* under constitutive promoters, in combination with the endoglucanase *eg2* (*T. reesei*) and β-glucosidase *bgl1* from *A. aculeatus*. Cultures were cultivated on acid- and alkali-pre-treated corncob-containing media, and the highest ethanol titres obtained within 7 days were 18.6 g/L [18]. 

Cell-surface display of cellulolytic enzymes has been demonstrated in *S. cerevisiae* using the glycosylphosphatidylinositol anchoring system [97]. This was achieved by incorporating a novel signal peptide sequence from the *S. cerevisiae SED1* gene onto *A. saculeatus* β-glucosidase (BGL1) and *T. reesei* endoglucanase II (EGII). Both secreted and cell-associated BGL1 and EGII were detected, showing higher levels (up to 1.9-fold activity) than using more conventional signal tags from enzymes glucoamylase (*Rhizopus oryzae*) and α-mating pheromone (*S. cerevisiae*). Ethanol titres of these constructs were up to 8.9 g/L when cultivated on cellobiose for 8 h [97].

An alternative to cell-surface display in *S. cerevisiae* is the production of trifunctional minicellulosomes [98]. The minicellulosomes were constructed using a miniscaffoldin containing a cellulose-binding domain and three cohesin modules, which were tethered to the cell surface through the yeast α-agglutinin adhesion receptor. Up to three types of cellulases were included, namely an endoglucanase, a cellobiohydrolase, and a β-glucosidase, each containing a C-terminal dockerin. Successful minicellulosome formation was dependent on the expression of the miniscaffoldin. These trifunctional complexes showed enhanced enzyme–enzyme and enzyme proximity synergy, and allowed the yeast to degrade and ferment phosphoric acid-swollen cellulose to ethanol (~1.8 g/L) [98]. 

Minicellulosomes have also been generated in bacterial systems, such as in the butanol-producing bacterium *Clostridium acetobutylicum* [99]. The cellulolytic genes *Cel9G*, *Cel48F*, and *Xyn10A* from *C. cellulolyticum* were integrated into the *C. acetobutylicum* genome with a miniscaffoldin derived from *C. cellulolyticum* CipC. Cellulosome anchoring was achieved using the native sortase system. The engineered strain demonstrated improved ability to grow on xylan as a sole carbon source with increased butanol titres, although no growth on cellulose polymers was observed [99]. 

### 3.3. Model Organism: E. coli

One of the most extensively utilised microbial chassis for bioengineering development is the bacterium *E. coli*. This is due to the development of an extensive genetic toolbox for manipulating its genome and transcriptome [100], and a detailed understanding of its endogenous metabolic pathways and regulation is available [101]. Steady-state metabolic flux models, such as EcoCyc, can predict the effects of gene knockouts and varying nutrient conditions [102], which are a useful tool for optimising strains for industrial applications. *E. coli* also possesses physiological properties highly desirable in an industrial host, such as fast growth kinetics [103], high levels of intracellular recombinant protein production [104], growth under both aerobic and anaerobic conditions [105], and use of a wide range of carbon sources including both C_5_ and C_6_ sugars [106]. The commercialisation of model organism *E. coli* as a microbial chassis is demonstrated in the production of insulin [107] and 1,3-propanediol [108]. 

Initial “proof-of-principle” pathway engineering and testing is commonly performed using *E. coli* prior to transitioning into more industrially relevant hosts. Examples of biotechnological routes to chemical production developed in *E. coli* are summarised in Table 1. The wide range of secondary products generated by engineered *E. coli* include synthetic fuels (primary and advanced), bioplastic monomers, flavours and fragrances, platform chemicals and pharmaceutical drug intermediates [23]. 

Previous attempts to endow *E. coli* with cellulolytic capabilities have focused on targeting specific secretory mechanisms, or in some cases the exploitation of chance discoveries (Table 2). These have included producing secreted soluble enzymes [123], cell-surface display [124] and the upregulation of naturally secreted “cryptic” cellulases in *E. coli* [125]. In each case, the major challenge was to overcome the barrier of cellulase secretion beyond the periplasmic space. This involves screening a variety of (typically) Gram-positive bacterial N-terminal signalling tags that have been proven to enable recombinant protein secretion in Gram-negative bacteria. 

The role of fusion partners in natural protein secretion in the laboratory strain *E. coli* BL21(DE3) was established by examining its extracellular proteome [127]. The most efficient fusion partner was OsmY, with titres of 250–700 mg/L of the target proteins alkaline phosphatase (*E. coli*), α-amylase (*B. subtilis*) and human leptin under high cell density cultivation. A later study used the OsmY-fusion protein approach to secrete β-glucosidase (*Cellvibrio japonicus*), endoxylanase (*Clostridium stercorarium*) and xylobiosidase (*C. japonicus*) from *E. coli* [123]. A co-culture of cellulolytic and hemicellulolytic strains successfully grew on ionic liquid pre-treated switchgrass (Table 2). These strains were subsequently engineered to produce fuel substitutes or precursors suitable for petrol, diesel and jet engines. For example, cultures grown in media containing 3.9% ionic liquid pre-treated switchgrass yielded 1.7 ± 0.6 mg∕L pinene. Improvements in both biofuel synthesis titres and lignocellulose digestion efficiencies could lead to the development of an economical route to advanced synthetic fuels [123]. 

The catalytic domain of cellulase Cel-CD from *Bacillus* sp. Z-16 was demonstrated to be efficiently secreted from *E. coli* to high levels (514 mg/L) in the absence of any known N-terminal signalling tag (Table 2) [127,128]. However, the N-terminal twenty amino acid sequence was found to be useful as a signalling tag to support the extracellular localisation of recombinant proteins in *E. coli*. For example, cellulose-hydrolysing strains of *E. coli* were engineered by fusing either Cel-CD or its N-terminal sequence to the β-glucosidase gene from *T. fusca* [126]. Further engineering was performed to incorporate a poly-3-hydroxybutyrate (PHB) synthesis pathway. This strain yielded 2.6–8.2 wt% PHB from cultures grown on amorphous cellulose and cellobiose, respectively. Two endoxylanases were also efficiently secreted into the culture medium when expressed with the N-terminal tag or a Cel-CD fusion [126].

Cell-surface display of cellulases on the *E. coli* LY01 outer membrane has been achieved by utilising the cell surface anchor PsgA from *B. subtilis* (Table 2) [129]. The *C. cellulolyticum* endoglucanase (Cel5A), exoglucanase (Cel9E) and β-glucosidase were surface displayed, allowing the strain to directly ferment dilute acid pre-treated corn stover to ethanol at 0.3 g/L. Higher titres were achieved from growth on phosphoric acid-swollen cellulose (3.6 g/L) [124]. 

A strain of *E. coli* has been isolated from bovine rumen that was capable of fermenting corn straw directly to both ethanol and hydrogen gas (Table 2) [125]. This strain was found to excrete cellulases with quantifiable exoglucanase, endoglucanase and β-glucosidase activities. Secondary product titres of 0.36 g/L ethanol and 4.71 mL/g hydrogen were achieved from growth on corn straw, with a cellulose/hemicellulose degradation ratio of 14.3%/11.4% [125]. Therefore, native *E. coli* strains exist with natural cellulolytic capabilities, which could potentially be exploited for secondary product generation with further engineering to increase growth rates on lignocellulose carbon sources.

These studies demonstrate the possibility of endowing cellulolytic properties on *E. coli* with secondary product titres, albeit at a reduced growth rate. In order for CBP to become a commercial reality, both increases in target compound titres and more efficient utilisation of lignocellulose waste need to be significantly improved. These latter gains could be made through the use of more efficient cellulases, improved extracellular secretion, higher levels of enzyme synergy, secretion and/or display.

## 4. Looking to the Future: The Biofoundry Approach

The power of the synthetic biology revolution is the ability to bypass traditional chemical syntheses and transition towards “natural” or biological production of commercially useful compounds. In the past few years, there has been an emergence of the biofoundry concept, which combines the strengths of the latest synthetic biological techniques with automation and high-throughput methodologies [130] to rapidly increase the rate at which biosynthetic pathways can be developed [131]. This emerging field of engineering biology focuses on the rapid design, build and testing of recombinant, industrially relevant microbial chassis, with the aim of generating new microbial strains for the sustainable and renewable production of chemicals, fuels and pharmaceuticals. 

Like any assembly line, final product selection is dependent on the set of “parts” utilised to build the biosynthetic pathway. These can include the choice of enzyme(s) homologue/variant, transcriptional/translational control, vector backbone [132] and DNA assembly techniques [132]. Therefore, optimal “parts” selection is critical for building efficient biofoundries, with more complex multi-step pathways requiring considerable optimisation screening to identify the best combination of parts for efficient production. To minimise the optimisation process, biofoundries typically employ integrated infrastructures that implement iterative Design-Build-Test-Learn (DBTL) cycles. This relies on automated pathway design and synthesis coupled to high throughput robotic screening. This then informs further design steps to improve process efficiency [133]. Automated design steps make use of retrosynthetic pathway design tools such as RETROPATH 2.0 [134], Selenzyme [135], PathPred [136], SimPheny [20] and SelProm [137]. For example, PathPred is a web-based server that designs bespoke biocatalytic pathways, and predicts which enzymes are suitable for each catalytic step [136]. This can be followed by Selenzyme, a free online tool that provides bespoke enzyme sequence selection for each biosynthetic step [135]. It uses existing databases to rank homologues of known biosynthetic abilities, based on conserved amino acid sequence regions, predicted active site, predicted soluble expression and transmembrane regions and the phylogenetic distance between the source organism and the target chassis [135]. The optimisation of promoter type and strengths is also critical for efficient pathway design. SelProm assists this design by acting as a parts repository for predictable plasmid expression strengths, using machine learning to select the best promoter systems to eliminate bottlenecks through biosynthetic pathways.

De novo robotic workflows and robotic technology platforms are key to biofoundry development as they can rapidly generate libraries of potential pathways and generate comparative performance data to enable the identification of the most suitable parts for target compound production. Machine-learning techniques are an integral part of the “Learn” step, as information from initial datasets can help make more informed decisions in future rounds of pathway design and screening dataset size [138]. 

The biofoundry approach has been demonstrated using a “pressure test” at the MIT-Broad foundry, where the challenge was to produce novel pathways to 10 compounds in just 90 days [139]. They successfully generated microbial chassis or cell-free systems for producing six out of the 10 targets (or a closely related molecule). Compounds produced included 1-hexadecanol, and the antifungal and antibacterial agents pyrrolnitrin and pacidamycin D, respectively. Novel routes were established towards the enediyne warhead underlying powerful antimicrobials, and a precursor to the chemotherapy agent vincristine was produced. In the case of tetrahydrofuran and barbamide production, in vivo pathway expression led to cytotoxicity, effectively halting further developments [139].

A similar rapid prototyping of microbial cell factories challenge by SYNBIOCHEM was performed to benchmark the capabilities of a biomanufacturing pipeline. They successfully produced 17 potential material monomers and key intermediates out of 25 identified compounds within 85 days [140]. This was performed by combining 160 genetic parts into 115 unique biosynthetic pathways. Compound classes targeted included vinylbenzenes, allylbenzenes, mandelate lactides and isobutyl compounds. The scale-up potential of these pathways was tested by optimising the enantioselective pathways to mandelic acid and hydroxymandelic acid. This generated gram-scale production titres using fed-batch fermentation. These studies show the power of these emerging biofoundries to quickly identify and implement novel pathways, albeit at “proof of principle” titres. These activities are beginning to be coordinated through the establishment of a Global Biofoundry Alliance [131]. 

Beyond proof of principle research, the biofoundry approach is a valuable tool when considering scaled processes. Biofoundaries could be implemented for pathway redesign and/or microbial chassis optimisation to increase chemical titres to be commercially competitive with existing natural and synthetic routes. In the case of CBP technologies, this approach could also be exploited to increase the currently slow rate of glucose release from cellulose, which impacts on the growth rates of the microorganisms. Key “parts” to be optimised could include maximising the synergism between (hemi)cellulolytic homologues, improving cellular excretion by de novo design of secretion tags and varying the relative transcriptional/translational rates necessary to generate the most efficient cellulolytic pathways. This is of particular importance when utilising (Gram-negative) bacterial chassis, where active cellulase secretion is severely limited. 

Successful implementation of the biofoundry approach for CBP requires efficient and sensitive high throughput screening techniques to ensure a sufficient library size can be sampled to maximise the chance of finding improved variants. Examples include the scaling of the International Union of Pure and Applied Chemistry (IUPAC) standardised methods for the detection of reducing sugars to a microtitre plate scale [141]. Other methods have been developed which allow the quantification of activity from whole cell preparations [142]. Coupled-assays based on the hydrolysis of fluorescent tags from 4-nitrophenyl β-D-glucopyranoside [143] and 4-nitrophenyl β-D-cellobioside [144] could also be scaled down to work in high throughput assays. Ultrahigh throughput screening techniques have been developed for cellulases. In vitro flow cytometry was used to screen exoglucanase variants generated through directed evolution using fluorescence from the hydrolysis of fluorescein-di-β-D-cellobioside [145]. Screens based on both fluorophore release [146] and coupled-assays [147] have been developed for use in microfluidics from whole cell samples. Bioprospecting techniques have also been used on the ultrahigh throughput scale [148]. The tools necessary to engineer novel cellulolytic pathways using biofoundries already exist, and they may hold the key to future successes in CBP. 

## 5. Conclusions

The often unacknowledged or underplayed bottleneck to the commercialisation of a synthetic biology generated microbial biomanufacturing process is the need to replace pure glucose-based carbon sources with cost-effective alternative feedstocks. Lignocellulose appears to be an ideal low-cost abundant waste feedstock, provided the extensive network of polymeric fibres can be degraded to release the abundant sugars contained within. However, the enzymatic degradation of lignocellulose is a complex process requiring thermochemical and/or mechanical pre-processing followed by the synergistic action of a variety of saccharolytic and lignin-degrading enzymes. The incorporation of an efficient cellulolytic system within microbial systems can be hampered by bottlenecks such as cost-effective lignocellulose pre-processing, lack of expression of functional glycosylated fungal cellulases in bacteria and the often-poor secretion of all the required (hemi)cellulases. 

The light on the horizon is the advent of the biofoundry approach, whereby traditional synthetic biology pathway design is replaced by iterative Design-Build-Test-Learn cycles. Between automated pathway design and synthesis, high throughput screening and machine learning, the process from concept to functionally efficient cellulolytic and high titre chemical producing industrial chassis might be realised in a relatively short time (months rather than years). The future outlook in the CBP field will therefore depend on the continued development of efficient automated and tunable engineering platforms, to accelerate both fundamental and applied biotechnological research to realise commercially successful biomanufacturing platforms.

## Figures and Tables

**Table 1:** Examples of compounds produced using engineered biosynthetic pathways in *E. coli.*

Product	Use	Design	Yield	Ref.
1,3-Propanediol	PTT production ^1^	Glycerol-3-phosphate dehydrogenase (DAR1 and GPP2) from *S. cerevisiae*. Glycerol dehydratase (dhaB1, dhaB2 and dhaB3) from *Klebsiella pneumoniae.* Endogenous ene-reductase (YqhD).	130 g/L	[108]
1,4-Butanediol	Advanced biofuel Polymer	Succinate semialdehyde dehydrogenase from *E. coli* and *Porphyromonas gingivalis.* 4-hydroxybutyrate dehydrogenase and 4-hydroxybutyryl-CoA transferase from *P. gingivalis.* Alcohol dehydrogenase from *Clostridium acetobuylicum.*	20 g/L	[20]
Ethanol	Biofuel	Pyruvate decarboxylase and alcohol dehydrogenase from *Z. mobilis.*	46 g/L	[109,110]
Isobutanol	Advanced biofuel	Endogenous 2-hydroxy-3-ketol-acid reductoisomerase, dihydroxy-acid dehydratase and alcohol dehydrogenase. Acetolactate synthase from *B. subtilis.* Ketoisovalerate decarboxylase from *L. lactis.*	22 g/L	[111]
Hydrocarbon gases (bio-LPG)	Advanced synthetic fuels	Multiple de novo metabolic routes based on amino acid utilisation, fatty acid biosynthesis, Clostridial butanol production and single step from butyric acid via fatty acid photodecarboxylase.	30–180 mg/g/d ^2^	[112,113]
(+)-Dihydrocarvide	Bioplastics	*Mentha spicata* route to carvone with an ene-reductase and cyclohexanone monooxygenase variant.	6.6 mg/L	[114]
Linalool	Hygiene products; chemical intermediate	“Plug-and-play” monoterpenoid production platform with linalool synthase.	363 mg/L ^3^	[28,29]
Fatty acid esters	Biodiesel	Thioesterase (*tesA*) and wax-ester synthase. Pyruvate decarboxylase and alcohol dehydrogenase from *Z. mobilis.*	674 mg/L	[115]
Limonene	Platform chemical Pharmaceutical industry	Heterologous methylerythritol 4-phosphate (MEP) pathway. Limonene synthase from *Mentha spicata*.	430 mg/L	[116,117]
Naringenin	Pharmaceutical industry	Flavanone pathway from L-tyrosine.	199 mg/L	[118]
Isopropene	Synthetic rubber	Heterologous mevalonate (MVA) pathway. Isoprene synthase from *Populus alba* and *P. kudzu*.	60 g/L	[119,120]
Taxiden-5α-ol	Taxol (anti-cancer drug)	Heterologous MEP pathway. Taxidene synthase from *Taxus brevifolia*, taxadiene 5α-hydroxylase and cytochrome P450.	58 mg/L	[16]
Succinic acid	Tetrahydrofuran	Knockdown of metabolic pyruvate drains. Pyruvate carboxylase from *Rhizobium etli.*	99 g/L	[19]
Hydrocodone	Opiate	Thebaine 6-O-demethylase and morphinone reductase from *Pseudomonas putida* and (*R*)-reticuline biosynthesis.	2.1 mg/L	[121,122]

^1^ Polytrimethylene terephthalate; ^2^ 30–180 mg propane per g cells per day; ^3^ Linalool titres are mg/L organic overlay, equivalent to 73 mg/L culture.

**Table 2:** Engineered *E. coli* to facilitate growth on lignocellulose carbon sources.

Feedstock	Cellulases	Export Tag	Product	Yield	Ref.
Ionic liquid pre-treated switchgrass	β-Glucosidase, endoxylanase and xylobiosidase	OsmY fusion	Fatty acid ethyl esters Butane Pinene	71 mg/L 8 mg/L 1.7 mg/L	[123]
Amorphous cellulose	Cel-CD and β-glucosidase	Cel-CD tag	3-hydroxybutyrate	0.3 g/L	[126]
Dilute acid pre-treated corn stover	Endoglucanase Cel5A, exoglucanase Cel9E, and β-glucosidase	PsgA	Ethanol	0.3 g/L	[124]
Corn straw	Endogenous cellulase	Native	Ethanol Hydrogen	0.36 g/L 3.3 mL/g	[125]

## Data Availability

No new data was reported in this manuscript.
